# Long-Term Consequences for Offspring of Paternal Diabetes and Metabolic Syndrome

**DOI:** 10.1155/2012/684562

**Published:** 2012-11-05

**Authors:** Benigno Linares Segovia, Maximiliano Gutiérrez Tinoco, Angeles Izquierdo Arrizon, Juan Manuel Guízar Mendoza, Norma Amador Licona

**Affiliations:** ^1^Division of Health Sciences, Department of Medicine and Nutrition, Guanajuato University, León, Mexico; ^2^Hospital Regional Salamanca, Petróleos Mexicanos, Salamanca, GTO, Mexico; ^3^Department of Vascular Surgery, Hospital Regional Salamanca, Petróleos Mexicanos, Salamanca, GTO, Mexico; ^4^Instituto Mexicano del Seguro Social, UMAE, León, Mexico

## Abstract

*Background*. Recent studies have reported an increase in the prevalence of obesity and metabolic syndrome in children and adolescents. However, few have focused how diabetes mellitus and metabolic syndrome together in parents can influence on obesity and metabolic disturbances in offspring. *Objective*. To know the risk obesity and metabolic disturbance in children, adolescents, and young adults whose parents have diabetes mellitus and metabolic syndrome. *Methods*. A comparative survey was made in healthy children of parents with diabetes mellitus and metabolic syndrome compared with offspring of healthy parents. We performed anthropometry and evaluated blood pressure, glucose, total cholesterol, HDL cholesterol, and triglycerides levels in plasma. We registered parent antecedents to diabetes mellitus and metabolic syndrome and investigated the prevalence of overweight, obesity, and metabolic disturbances in offspring. *Results*. We studied 259 subjects of 7 to 20 years of age. The prevalence of overweight and obesity was 27% and 37%, respectively. The highest proportion of BMI >95th of the entire group was found in offspring with both diabetic parents. Glucose and total cholesterol levels were lower in the group with healthy parents compared with the group with diabetic mother and metabolic syndrome but with healthy father. HDL cholesterol was higher in the group with both healthy parents than in the group with diabetic mother and metabolic syndrome but healthy father. *Conclusions*. The offspring of parents with diabetes plus metabolic syndrome showed higher proportion of variables related to metabolic syndrome compared with healthy parents.

## 1. Antecedents

Obesity is the most frequent nutritional disease in children and adolescents in developed countries. In 2009-2010, 16.9% of US children and adolescents were obese and nowadays Mexico has also increased the prevalence of obese children [1, 2]. Obesity has become a pandemic that affects to more than a trillion of the people in the world [3]. In adults, are well known the medical consequences associated with obesity (diabetes, cardiovascular disease, and hypertension) and their relationship with metabolic syndrome (MS). Several studies have reported an increased prevalence of obesity and MS in children and adolescents as well as how obesity and diabetes in parents may influence obesity and metabolic conditions in their offspring [4–12]. However, not all diabetic subjects show MS or all subjects with MS develop diabetes mellitus. Thus, the aim of this study was to determine the influence of history of diabetes, obesity, and MS in parents on the metabolic conditions of their offspring.

## 2. Methods

### 2.1. Study Population

The local Ethical Committee at the PEMEX Hospital approved the study, and informed consent was obtained from all participants and at least one parent.

We studied 259 families randomly selected from the clinic of cardiovascular and metabolic risk in the PEMEX Regional Hospital in Salamanca, México. In this clinic, adults usually are sent in case of BMI >25 and physicians try to rule out diabetes mellitus, MS, and dyslipidemia. After selecting parents with biological children, we randomly choose only one child between 7 and 20 years old per family to be included in the study. The criteria used to diagnose the MS in parents were taken from those of the National Cholesterol Education Program's Adult Treatment Panel, ATP III [13]. The parents' antecedent of diabetes mellitus, blood pressure, obesity, waist to hip index, cholesterol, HDL-cholesterol, triglycerides, and the MS diagnosis was obtained from the clinical file. 

The sample size was determined with the formula for proportions (42% for obesity in adults in the state of Guanajuato according to the National Survey of Health in the year 2000 [14]) according to confiability of 95%, unilateral *α* = 0.05, and *β* = 0.10. 

We distributed the group of parents as follows to compare characteristics in offspring: (1) both parents without diabetes or MS, (2) diabetic father (with or without MS) but healthy mother, (3) diabetic mother (with or without MS) but healthy father, (4) both parents with diagnosis of diabetes mellitus, (5) father with diabetes + MS but healthy mother, and (6) mother with diabetes + MS but healthy father.

### 2.2. Procedures

All the children and young adults were evaluated at 8:00 h, after fasting of ≥12 hours. Before rest of at least 15 minutes, we measured the blood pressure levels with a mercury sphygmomanometer whose bracelet covered the 2/3 parts in the right arm. In the younger children a pediatric-sized blood pressure cuff was used. We performed 2 readings with at least 5 minutes of difference and the average was registered for analysis.

They were stratified in 4 groups according to their age: (I) prepuber, of 7 to 10 years; (II) puber, from 11 to 14 years; (III) postpuber, of 15–17 years; (IV) young adult, of 18 to 20 years.

### 2.3. Anthropometric Measurements

Standard calibrated scales and stadiometers were used to determine height, weight, and BMI. Because BMI changes with age, BMI-for-age percentiles were calculated according to the Centers for Disease Control and Prevention growth charts [15], with the following equation: *C* = *M*(1 + *LSZ*)^2^(1/*L*), where *C* represents the age (in months) adjusted percentile for a given measurement (e.g., BMI), *M* represents the age-related median, *S* represents the age-related SD, *L* represents the age-related power in the Box-Cox transformation, and *Z* represents the *z* score. The age-adjusted *z* score corresponding to the exact percentile for a given measurement was calculated with the following equation: *Z* = [(*X*/*M*)^2^ *L* − 1]/*LS*, where *X* represents the physical measurement (e.g., weight, length, head circumference, stature, or calculated BMI value) and the *L*, *M*, and *S* values were again collected from the appropriate table corresponding to the age (in months) of the child. All measurements were performed by the same trained individual.

### 2.4. Biochemical Analysis

Plasma glucose levels were analyzed with an automated instrument (ALCYON 300/3001 Analyzer; Abbot Laboratories, intraassay coefficient of variation, 1.7%; interassay coefficient of variation, 7.5%). Serum total cholesterol, triglycerides (TG), and high-density lipoprotein (HDL) cholesterol levels were analyzed with an automated instrument (Synchron CX4 PTO Analyzer; Beckman Coulter, intraassay and interassay coefficient of variation was 3.0% and 4.5%, resp.). The studies were made in the laboratory of the PEMEX Regional Hospital in Salamanca, Mexico.

### 2.5. Definitions

Obesity was defined as a BMIz score ≥2.0, adjusted by age and sex. The subjects were classified as moderately obese (a BMIz score of 2.0 to 2.5) or severely obese (a BMIz score above 2.5). Elevated systolic or diastolic blood pressure was defined as a value that exceeded the 95th percentile for age and sex. Abnormalities in glucose levels, BMI, and lipid profile were considered in case of values ≥95th of the entire group. 

## 3. Statistical Analysis

Results are expressed as mean ± SD, as median (95% CI), or as a proportion according to variables' distribution. Differences between genders were assessed by *χ* ^2^ for proportions. ANOVA was used for continue variables and Tukey's honest significant difference was performed as post hoc test. A *P*  value <0.05 is considered significant.

## 4. Results

We studied 259 children and young adults distributed as follows: 47 (18.1%) prepuber, 60 (23.2%) puber, 86 (33.2%) postpuber, and 66 (25.5%) young adults. The prevalence of overweight and obesity was of 27% and 36.7%, respectively. There was no difference by age (*t* = 1.21, *P* = 0.26) or by gender (*P* = 0.23).

No difference was found in gender and age in offspring between groups of parents according to their metabolic state. BMI, SBP, and total cholesterol were higher in the group of diabetic father + MS but healthy mother than in the group with both healthy parents. Also BMI was higher in the case of father with diabetes without considering MS status but with healthy mother than in the group of healthy parents. Glucose levels and total cholesterol levels were lower in the last group compared with the group with diabetic mother and MS but with healthy father. HDL cholesterol was higher in the group with both healthy parents than in the group with diabetic mother and MS but healthy father (Table 1). 

Pubertal state was similar between the groups of parents. However, the highest proportion of BMI >95th of the entire group was found in case of both parents with diabetes while the highest proportion of cholesterol levels >95th of the entire group was found in the group with diabetic father + MS but healthy mother. Also these two groups showed the highest number of variables >95th of the entire group (Table 2).

## 5. Discussion

Several authors have studied the prevalence and predictors of overweight and metabolic disorders in offspring of mothers with gestational diabetes mellitus (GDM) or type 1 diabetes [16–19], other studies have focused on the effect of family history of type 2 diabetes mellitus (T2DM) on lipid and carbohydrate metabolism in children [20, 21]. In this study we analyzed the prevalence of overweight, obesity, and metabolic disorders in offspring of parents with diabetes mellitus with and without metabolic syndrome in relation to the offspring of parents without these metabolic disorders. We found the highest proportion of BMI >95th of the entire group in offspring with both diabetic parents. Supporting our results, Clausen et al. [17] reported an increased prevalence of overweight in adults who were from GDM pregnancies (40%) or type 1 diabetes (T1DM) pregnancies (41%) compared with control subjects (24%). Lawlor et al. [16] found only a minor increase in the prevalence of overweight in offspring of mothers with GDM (30%) compared with control children (23%) and offspring of mothers with T1DM (23%). It has been considered that higher BMI in offspring of GDM and offspring of mothers with T1DM compared to the control group may be due to relatively higher levels of insulin. Recently, a common variant in the *FTO* (fat, mass, and obesity) gene has been identified that predisposes to diabetes through an effect on the BMI. It was shown that the risk of high BMI and hence of a predisposition to diabetes was additive and that individuals homozygous for this particular SNP (allele A) had a higher BMI as compared to heterozygote individuals [22].

In our study, glucose and total cholesterol levels were lower in the group with healthy parents compared with the group with diabetic mother and metabolic syndrome but with healthy father. In accordance with our results, Srinivasan et al. report that the offspring with parental diabetes versus those without such history had significantly excess generalized and truncal adiposity beginning in childhood [11]. Like these authors, we observed higher levels of total cholesterol and lower HDL cholesterol levels in offspring of parents with T2DM compared with the offspring of nondiabetics; this difference was more significant when at least one parent was diabetic and also had metabolic syndrome. Although we did not document any case of diabetes in the offspring, the mean serum glucose in our series was significantly higher than 69.2 mg/dL reported by Lin et al. [23] as baseline the subjects of his study, and in which they describe that those patients, who progressed to diabetes, increased their levels at a rate of 2.27 mg/dL per year. Derived from this we would expect the presence of diabetes at earlier stages of life in our population.

## 6. Conclusions

The offspring of parents with diabetes showed higher proportion of variables related to metabolic syndrome compared with those offspring of healthy parents, and this proportion increased in case of diabetic parents plus metabolic syndrome.

## Figures and Tables

**Table 1:** Offspring characteristics according to antecedents of diabetes or metabolic syndrome in their parents compared with healthy parents.

Variable in offspring	Healthy father and mother *n* = 114 (1)	Diabetic father and healthy mother *n* = 45 (2)	Diabetic mother and healthy father *n* = 78 (3)	Both parents with diabetes *n* = 22 (4)	Diabetic father and MS, healthy mother *n* = 19 (5)	Diabetic mother and MS, healthy father *n* = 23 (6)	*P*	Post hoc
Gender M/F	52/62	24/21	37/41	9/13	11/8	11/12	0.85	—

Age (years)	14.8 ± 3.3	14.2 ± 4.0	14.6 ± 3.5	15.6 ± 3.8	14.0 ± 3.7	15.3 ± 3.1	0.45	—

BMI	24.7 ± 5.5	27.5 ± 6.3	24.8 ± 5.2	25.4 ± 6.1	27.4 ± 7.7	24.9 ± 5.8	0.05	1 versus 2 *P* = 0.02 1 versus 5 *P* = 0.05

SBP (mmHg)	98.2 ± 13.6	100.8 ± 15.0	96.5 ± 11.7	102.5 ± 11.9	105.7 ± 17.1	99.1 ± 14.4	0.02	1 versus 5 *P* = 0.01

DBP (mmHg)	64.1 ± 9.0	65.1 ± 10.9	63.4 ± 9.3	65.0 ± 10.1	66.3 ± 12.5	66.0 ± 12.3	0.62	—

Glucose (mg/dL)	89.9 ± 11.1	93.2 ± 8.0	93.8 ± 11.9	92.3 ± 10.3	93.5 ± 7.8	95.8 ± 17.1	0.01	1 versus 6 *P* = 0.01

Total cholesterol (mg/dL)	150.9 ± 29.9	156.9 ± 29.7	160.4 ± 37.4	158.5 ± 37.4	168.3 ± 27.5	165.6 ± 44.3	0.004	1 versus 5 *P* = 0.01 1 versus 6 *P* = 0.03

HDL-chol	66.0 (60.5–70.5)	68.0 (57.5–70.5)	62.0 (53.0–66.5)	67.2 (46.5–74.0)	62.5 (39.6–78.5)	47.0 (41.0–66.5)	0.05	1 versus 6 *P* = 0.04

Triglycerides (mg/dL)	104.0 (94.0–116.0)	103.0 (82.0–136.0)	109.5 (88.0–129.0)	128.0 (85.0–140.0)	136 (80.0–61.0)	132 (89.0–160.0)	0.34	—

**Table 2:** Proportion of puberal state, values >95th of the entire group, and number of abnormalities according to antecedents of diabetes or metabolic syndrome in their parents compared with healthy parents.

Variable in offspring	Healthy father and mother *n* = 114 (1)	Diabetic father and healthy mother *n* = 4 (2)	Diabetic mother and healthy father *n* = 78 (3)	Both parents with diabetes *n* = 22 (4)	Diabetic father and MS, healthy mother *n* = 19 (5)	Diabetic mother and MS, healthy father *n* = 23 (6)	*P*
Pubertal stage *n* (%)							
Prepuber	18 (15.8)	11 (24.4)	13 (16.6)	5 (22.7)	5 (26.3)	3 (13.0)	0.41
Puber	27 (23.6)	10 (22.2)	23 (29.5)	0 (0)	4 (21.1)	4 (17.4)	
Postpuber	41 (35.9)	14 (31.1)	22 (28.2)	9 (40.9)	8 (42.1)	11 (47.8)	
Young adult	98 (87.9)	10 (22.2)	20 (25.6)	8 (36.4)	2 (10.5)	5 (21.7)	
Variables >95th *n* (%)	114	45	78	22	19	23	
BMI	48 (42.1)	31 (68.9)	34 (43.6)	11 (50.0)	11 (57.9)	9 (39.1)	0.04
SBP	46 (40.3)	22 (48.9)	34 (43.6)	12 (54.5)	8 (42.1)	12 (52.2)	0.84
DBP	46 (40.3)	21 (46.6)	33 (42.3)	11 (50.0)	8 (42.1)	12 (52.2)	0.81
Glucose	34 (29.8)	22 (48.9)	34 (43.6)	10 (45.4)	9 (47.4)	12 (52.2)	0.11
Total cholesterol	41 (35.9)	23 (51.1)	38 (48.7)	11 (50.0)	14 (73.7)	12 (52.2)	0.03
HDL-Chol	32 (28.1)	12 (26.7)	29 (37.2)	7 (31.8)	8 (42.1)	12 (52.2)	0.20
Triglycerides	48 (42.1)	21 (46.6)	38 (48.7)	14 (63.6)	12 (63.1)	12 (52.2)	0.33
Number of variables <95th	2 (2-3)	4 (3-4)	3 (2-3)	3.5 (2–5)	4 (2–4)	3 (2–5)	0.009
